# Epidemiology of head injuries in pedestrian-motor vehicle accidents

**DOI:** 10.1038/s41598-023-47476-z

**Published:** 2023-11-20

**Authors:** Behzad Zohrevandi, Enayatollah Homaie Rad, Leila Kouchakinejad-Eramsadati, Ghazaleh Imani, Iman Pourheravi, Naema Khodadadi-Hassankiadeh

**Affiliations:** 1 Guilan Road Trauma Research Center, Trauma Institute, Guilan University of Medical Sciences, Rasht, Iran; 2 Social Determinants of Health Research Center, Trauma Institute, Guilan University of Medical Sciences, Rasht, Iran; 3 School of Medicine, Guilan University of Medical Sciences, Rasht, Iran

**Keywords:** Neuroscience, Anatomy, Health care, Medical research

## Abstract

Despite efforts of many countries to improve pedestrian safety, international reports show an upward trend in pedestrian-motor vehicle accidents. Although the most common cause of death of pedestrians is head injuries, there is a lack of knowledge on the epidemiology and characteristics of head injury in terms of the Glasgow Outcome Scale to be used for prevention. However, this study aimed to determine the epidemiology of pedestrian-motor vehicle accidents, the characteristics of head injury, and differences in the Glasgow Outcome Scale in terms of gender. In this retrospective analytical study, the data of 917 eligible injured pedestrians were obtained from the two databases of the Trauma System and the Hospital Information System. The data were analyzed using SPSS software (Version 21). The mean age of all 917 injured pedestrians was 47.55 ± 19.47 years. Most of the injured pedestrians (42.10%) were in the age range of 41–69 years and 81.31% were male. Moreover, 83.07% did not have any acute lesions on the CT scan. The most common brain lesion was brain contusion (n = 33, 3.60%), subarachnoid hemorrhage (n = 33, 3.60%), and skull fracture (n = 29, 3.16%). Among all concurrent injuries, lower extremity/pelvic injuries were observed in 216 patients (23.56%). Outpatient treatment (n = 782, 85.27%), airway control/endotracheal intubation (n = 57, 6.22%), and resuscitation (n = 35, 3.82%) were the most applied treatments respectively. There were significant differences in the Glasgow Outcome Scale between men and women (*P*- value = 0. 012). The high rate of mortalities, disability, head injuries, contusion, subarachnoid hemorrhage, and skull fractures in pedestrians involved in MVAs emphasizes the need for developing and implementing prevention strategies including appropriate management and risk reduction. Male pedestrians were at higher risk of motor vehicle accidents and worse Glasgow Outcome Scale. The presented data identified the main types of pedestrian injuries and suggested the importance of adopting appropriate preventive strategies to achieve the most effective interventions for creating a safer community.

## Introduction

Pedestrians are considered the most vulnerable road users. Significant differences between the injuries sustained and injury risk by males and females were obtained^1,2^.

In 2018, 6227 pedestrians were killed in pedestrian-motor vehicle accidents (PMVAs), accounting for about 15% of all MVAs in the United States^3^. In Canada, about 25% of injuries occurred to young pedestrians due to these accidents^4^. Despite efforts of many cities in the United States to improve the safety of pedestrians, national statistics show an upward trend in the number of pedestrian accidents. In 2017 and 2018, pedestrian casualties increased by 4% and 1.7%, respectively. The highest number of fatalities since 1990 belonged to the year 2018^3^. Mitchell and Bambach (2016) reported that in the study of 9781 pedestrians injured in traffic accidents, in terms of cost, a total of 2.4 billion dollars in personal injury recovery costs and an annual cost of 243 million dollars were estimated. In their scrutiny, 22.6% of total injury recovery costs were allocated to TBI. Over one-third of pedestrians had 4 simultaneous injuries, with an average cost of $243,992, which was 1.6 times more than the cost of a pedestrian with only one injured part^5^.

The rate of severe injuries and consequent mortalities of pedestrians is increasing in Iran^6^. According to the Iranian Legal Medicine Organization, pedestrian deaths accounted for about one-fifth (19.8%) of all deaths due to PMVAs, suggesting an increase compared with the past. The highest rates of pedestrian mortalities concerning the total mortalities due to traffic accidents belonged to Tehran (38.6%), Mazandaran (35.4%), and Guilan (32.7%) provinces, respectively^7^.

Pedestrian MVAs often lead to deaths or severe injuries and comprise a large proportion of the socio-economic damages caused by traffic accidents. Thus, a significant level of safety needs to be provided for pedestrians and effective countermeasures should be taken to reduce the number of vehicle accidents involving pedestrians. Understanding the pattern of PMVAs is important because the information and results obtained are really useful for the future treatment of head injuries in pedestrians. This ultimately helps us propose the most effective preventive measures^8–10^.

Traumatic brain injury is a major problem that can lead to short-term symptoms such as dizziness, memory loss, blurred vision, and loss of consciousness (LOC). The most common cause of death of pedestrians involved in accidents is concussion^11^. Even after recovering from concussion, patients may suffer from long-term neurological and psychological problems which are often referred to as post-traumatic symptoms (syndrome)^12^.

In a study on pedestrians who visited emergency departments in North Carolina over a five-year period, the injury pattern was determined by age. People aged 0–14 years had the highest proportion of head injury (39.5%) and adults over 65 years had the highest proportion of spine/vertebrae (12.6%) and upper extremity injuries (33.2%). In terms of the nature of the injury, people aged 0–14 had the highest percentage of TBI (11.4%) and superficial wounds and contusions (62.8%). Adults over 65 years old had the highest ratio of wound/amputation and open fracture (16.1%). Adults aged 25–64 had the highest proportion of strains/sprains/dislocations (18.7%)^13^. In a study in Southwest China investigating pedestrian accidents, the most common age group involved was between 51 and 80 years old, and pre-hospital mortality was reported in 371 cases (56.64%). Skull fracture was observed in 55.60% of cases and the most common fractured bone was the temporal bone. The most common type of intracranial injury was subarachnoid hemorrhage^14^.

The Glasgow Coma Scale (GCS), developed in 1974 by Teasdale and Jennett, is the most widely used behavioral measure to assess the severity of acute TBI. It is used in almost all clinical and research contexts to measure the initial severity of a TBI due to its simplicity and rapid assessment approach. Although it is internationally accepted for the diagnosis and prognosis of TBI, it may not accurately reflect the level of consciousness^15–17^. The Glasgow outcome score (GOS) is a tool for neurological assessment of TBI patients, used both at hospital discharge (retrospective), and at least one year after TBI (prospective). Based on the GOS, TBI patients are classified into five categories: dead, vegetative state, severe disability, moderate disability, and good recovery^18,19^.

Although most studies have shown that pedestrians are exposed to a higher risk of death than other road users, there is limited knowledge of the epidemiology of pedestrian MVAs, the characteristics of their head injuries, and different GOS in terms of gender to be used for prevention. This study analyses pedestrian TBI which is not only an important topic in researching traffic safety but also is interesting for practitioners in public health and surgery because it presents characteristics of head injuries in pedestrians in Iran. Therefore, the present study determines the epidemiology of PMVAs, head injury characteristics, and gender differences in GOS of pedestrians hospitalized in a referral hospital in the north of Iran.

## Material and methods

This retrospective analytical study was conducted after receiving an ethical code from the Ethics Committee of Guilan University of Medical Sciences. The requirement for informed consent from the study subjects was waived by the ethics committee of Guilan University of Medical Sciences, Rasht, Iran (IR.GUMS.REC.1399.179) due to the retrospective study design. The researchers of this study confirm that all methods were performed in accordance with the relevant guidelines and regulations. The setting of this study was Poursina Hospital, a tertiary trauma center with the highest number of referrals in Guilan province located in the north of Iran. Rasht is the capital of Guilan province with many counties and rural areas. All patients injured in minor to major road accidents in Rasht and the neighboring areas are transferred to this center. However, referrals from other cities only include severely injured and critically ill patients.

All injured pedestrians involved in PMVAs who were admitted to Poursina Hospital were included in the study provided they met the entry criteria.

Inclusion criteria were as follows: (a) The PMVAs occurred in the Guilan province period between May 2017 and April 2020 (b) The pedestrians who were over 15 years old.

Pedestrians whose available data in the Trauma System and HIS were very incomplete were excluded from the study.

The data required for this study were collected from two sources:

1. Trauma system database: These data were obtained in an Excel file with the permission of the Statistics and Medical Records of the Trauma System Unit.
2. Hospital Information System (HIS): Variables such as CT scan findings of these patients (epidural hemorrhage, subdural hemorrhage, intraparenchymal hemorrhage, cerebral contusion, sunken fracture, skull base fracture, etc.) not existing in the trauma system database were obtained by record reading in HIS.

Two medical students (interns) were trained in how to collect data. First of all, they extracted collision data in an Excel file from the trauma system database. All of the data registered in the trauma system from the beginning (May 2017–April 2020) including 917 eligible subjects were entered into the analysis. Using patient ID, the information of the same sample was obtained from the patient's medical records on HIS.

## Measure

The data required for this study were collected by three questionnaires:

A. Demographic characteristics: Age, sex, and occupation
B. Geographical & transfer characteristics: Residency, collision location, collision area, mode of transfer to hospital
C. Clinical characteristics: Blood pressure, heart rate, oxygen saturation, trauma type, GSC (mild GCS 13–15, medium (GCS 9–12), and severe (GCS 3–8)^20,21^, initial symptoms after the head injury, hospital therapies, surgery type, CT scan findings (epidural hemorrhage, subdural hemorrhage, intracranial hemorrhage, intraparenchymal hemorrhage, contusion, and skull fracture, etc.) concurrent injuries, and GOS at the time of discharge obtained from Trauma System database retrospectively (dead, vegetative state, severe disability, moderate disability, and good recovery)^18,19^ at discharge based on trauma system database. Chi-squared test was used to examine the relationship between gender variables and GOS. Analyses were performed using SPSS software (version 21) and *p*-value < 0.05 was considered as the significance level.

## Results

The mean age of all injured pedestrians was 47.55 ± 19.47 years. Most of them (n = 388, 42.10%) were in the age range of 41–69 years. Of all 917 participants, 746 (81.31%) were men and 298 (32.47%) were self-employed (Table 1).

**Table 1:** Demographic characteristics of pedestrians involved in PMVA (n = 917).

Variable	Categories	N (%)
Age	15–40	383 (41.95)
	41–69	388 (42.10)
	≥ 70	146 (15.95)
Gender	Male	746 (81.31)
	Female	171 (18.69)
Occupation	Self-employment	298 (32.47)
	Employee	132 (14.43)
	Unemployed	99 (10.80)
	Retired	45 (4.91)
	Housekeeper	253 (27.57)
	Student	81 (8.83)
	Others	9 (0.99)

The highest number of PMVAs occurred in residents of Rasht (780, 80.4%) on Alleys & streets (830, 91.53%), and in urban areas (778, 82.80%). Almost half of the injured pedestrians (454, 52.40%) were transferred to the hospital by the Emergency Medical System (EMS) (Table 2).

**Table 2:** Geographical & transfer characteristics of the PMVAs (n = 917).

Variable	Categories	N (%)
Residency	Capital (Rasht)	780 (80.4)
	Other cities	137 (19.6)
Collision location	Alleys & streets	830 (91.53)
	Roads & highways	71 (6.97)
	Leisure & sport centers	12 (1.13)
	School	1 (0.09)
	Workplace	1 (0.09)
	Public places	2 (0.19)
Collision area	Urban	778 (82.80)
	Rural	129 (16.15)
	Suburb	10 (1.05)
Mode of transfer	EMS	454 (52.40)
	Inter-hospital transfer	34 (4.69)
	Private car	429 (42.91)

On admission, the mean systolic blood pressure was 122.17 ± 21.97, and the mean diastolic blood pressure was 73.01 ± 12.48. The mean heart rate was 92.10 ± 45.22, and the mean oxygen saturation was 96.55 ± 4.24.

Most of the patients (n = 476, 50.41%) suffered from penetrating trauma. GSC of the majority of PMVAs was fully conscious (n = 855, 94.03%) and three of the most common initial symptoms after head injury were headache (n = 167, 18.21%), nausea/vomiting (n = 157, 17.12%) and head/face injury (n = 132, 14.29%), respectively. Three of the most common therapies performed in the hospital were outpatient treatment (n = 782, 85.27%), airway control, and endotracheal intubation (n = 57, 6.22%) and resuscitation (n = 35, 3.82%) respectively. The prevalence of lesions based on CT scan results showed that the majority of the pedestrians (n = 762, 83.07%) had no acute pathological lesions. Three of the most common pathological finding was brain contusion (n = 33, 3.60%), subarachnoid haemorrhage (SAH) (n = 33, 3.60%), and skull fracture (n = 29, 3.16%). The most common concurrent injuries were lower limb/pelvic injuries (n = 216, 23.56%) (Table 3).

**Table 3:** Clinical characteristics of pedestrians (n = 917).

Variable	Categories	N (%)
Trauma type	Blunt	441 (49.59)
	Penetrating	476 (50.41)
GCS	Mild	855 (94.03)
	Medium	23 (2.30)
	Sever	39 (3.67)
Initial symptoms after head injury	Headache	167 (18.21)
	Nausea & vomiting	157 (17.12)
	Head & face injury	132 (14.29)
	Vertigo	69 (7.52)
	Rhinorrhea & Rhinorrhagia	22 (2.40)
	Otorrhea	10 (1.38)
	Raccoon sign	71 (7.74)
	Hemotympanum	33 (3.60)
	LOC	37 (4.03)
	Scalp lacerations	108 (11.78)
	Others	111 (11.93)
Hospital therapies	Outpatient treatment	782 (85.27)
	Airway & endotracheal intubation	57 (6.22)
	Resuscitation	35 (3.82)
	Bleeding & volume lost control	30 (3.27)
	Neurological Surgery	13 (1.42)
CT scan finding	No acute pathological	762 (83.07)
	Contusion	33 (3.60)
	SAH	33 (3.60)
	Skull fracture	29 (3.16)
	Epidural hemorrhage	14 (1.53)
	Subdural hemorrhage	17 (1.85)
	Intraparenchymal hemorrhage	21 (2.29)
	Other	8 (0.9)
Concurrent injury & condition	Lower limb & pelvic injury	216 (23.56)
	Upper limb injury	148 (16.14)
	Chest injury	43 (4.69)
	Abdominal injury	26 (2.84)
	Spinal cord injury	22 (2.40)
	Fracture and dislocation of the neck	9 (0.1)
	Hemorrhagic shock	3 (0.33)

In terms of GOS, the majority of men and women were discharged with “good recovery”. A total of 9 individuals developed severe disabilities after the PMVAs; eight were men and only one was a woman. The results were statistically different in men and women (*P*-value = 0.012) (Table 4).

**Table 4:** GOS of pedestrians involved in PMVA (n = 917).

GOS					
	Death	Vegetative state	Severe disability	Moderate disability	Good recovery
Male	36 (4.88)	1 (0.14)	8 (1.09)	73 (9.91)	619 (83.99)
Female	9 (5.26)	0 (0)	1 (0.58)	3 (1.75)	158 (92.40)

Pearson Chi-squared test = 12.786, *P*- value = 0. 012 *missing = 9.

## Discussion

The present study sought the epidemiology of pedestrian head injuries in northern Iran. Most brain CT scans indicated no lesions. Serious injuries were associated with factors such as speed limits, lighting condition, number of road lanes, and vehicle type^22^.

The three most common lesions detected were contusion, SAH, and skull fracture, respectively. In a similar study, skull fracture (23.9%) and intracranial hemorrhage (25.2%) accounted for about half of all pedestrian injuries^23^. The results can vary and be affected by the type of vehicle, vehicle speed, etc. This probably indicates that the pedestrians under study experienced less severity of the PMVAs with a lower average speed in urban areas, but this is not for certain as ISS (injury severity scores) was not checked. However, about 13% of pathological lesions were reported on their brain CTs. The majority (94%) of our patients had normal GCS, vital signs (blood pressure and heart rate), and arterial oxygen pressure, hence it can be claimed that most of the injuries were mild. In a similar study, superficial injuries accounted for 29.1% of the most common pedestrian injuries^24^.

In the present study, three of the most common initial symptoms after head injury that occurred at arrival time to the hospital included headache, nausea/vomiting, and head/face injury. In one study, only about 6–9% of patients with mild head trauma had intracerebral lesions and 0.4% of them required surgery^25^. In a simulator study, the head hits the top of the hood after the front hood strikes the left foot of the pedestrian. The pedestrian spins in the air and falls to the ground, making the first contact with the ground with his head and the second with his right shoulder. In short, the head of the pedestrian is hit several times in a collision^26^. Therefore, having simple early symptoms like headaches will be expected.

In the present study, lower limb and pelvic injuries followed by upper extremity and chest injuries were the three most prevalent injuries that occurred with head injuries simultaneously. In another study, the traditional triad of pedestrian injuries i.e. head, pelvis, and knee were introduced^27^. Other researchers reported that head injury, because of the high number of mild concussions, was the most common injury site in pedestrians. However, putting head injuries aside, the most common serious injuries were to lower limbs^28^. In a recent study, most of the injuries were reported in the legs followed by the head and neck, and damages to the upper extremities, respectively^4^. Different results can be related to a diverse range of factors, including different methods (heterogeneity in defining variables in HIS) used in hospital registration systems and the development of the automotive industry of countries. Li et al. (2019) have reported that large hoods used in producing new vehicles can cause more damage to the pelvic bone in the event of a pedestrian-vehicle accident, adding that using wide, flat bumpers in new vehicles can reduce lower limb fractures^29^. Depending on the type of vehicle, the consequences of injury to pedestrians can be different in developed and developing countries. Meanwhile, if new cars with safe hoods are produced in our country's car industry, the severity of pedestrians' lower injuries can be reduced. In the present study, the type of vehicle was not accurately recorded so the correlation between its weight with mortality and morbidity could not be explored. Moreover, children have different patterns of injuries from adults. In our study, children under ten years were also included. Therefore, the inclusion criteria for entering the current study were different from those of other studies in terms of age which obviously led to different findings.

The mortality rate in the present study was 4.91%, but in similar studies, a higher rate of pedestrian mortality was reported i.e. 23.2% in South Africa. Furthermore, according to hospital reports, head injuries were the leading cause of death among pedestrians. However, the details of the type of brain injury have not been reported^30^. In another study, the second most common cause of death in pedestrians after hemorrhage/shock (64.7%) was head injury (26.7%)^23^. Apart from the report on good vital signs of most pedestrians, another reason for the low mortality in the present study can be attributed to the fact that the majority of PMVAs occurred on streets of urban areas in Rasht city. Thus, the injury severity and mortality rate were lower than those of PMVAs occurring on roadsides and highways, where vehicles drive faster^6^.

In the present study, most of the pedestrians suffered from penetrating trauma and were transmitted to the hospital by EMS, although a significant number of patients had been transferred by those present at the scene. Nonetheless, in one study, the time to reach the scene and the rate of EMS transfer did not predict hospital mortality but ISS was a good predictor^31^.

The most common therapeutic measures in the present study were outpatient treatment, airway and endotracheal intubation, and resuscitation, respectively. In an epidemiological study on traumatic brain injury in Iran, control of bleeding and the lost volume were greater than other operations, and the rate of the other two treatments; airway control and endotracheal intubation, and resuscitation, were equal^32^. However, treatment measures by EMS such as airway management, control of bleeding, and the loss volume will play an important role in reducing mortalities and traumatic injuries^33–35^. EMS personnel have the information and knowledge to deal with these emergencies, but such conditions will not be provided in private transfer^36^.

In the present study, three of the most common surgeries performed for pedestrians with head injuries were scalp laceration repair surgery, lower limb surgery, and upper limb surgery, respectively. In a similar study, however, most pedestrian surgeries were related to the lower extremity. The majority of injuries in the subjects were scalp laceration and therefore, most of the surgeries were laceration repairs. If we do not consider this simple repair as surgery, then our findings would be exactly the same as those of Bogert's study on the same lower limb surgery in 2019^37^.

In the present study, the mortality rate, and the severe disability rate, were significantly higher in men than in women. In a similar study, the death likelihood was 2.3 times higher in men than in women^38^. The results of a study provide strong indications of an existing relationship between individual and social contexts of pedestrians and their involvement in MVAs, including the gender of the pedestrian. It was also found that male pedestrians were more exposed to PMVA^39,40^. Higher rates of mortality and morbidity in men can stem from the higher overall mortality of men than women and more presence in traffic^2,40^.

The majority of patients in the study were discharged from the hospital in good general condition, but moderate disabilities were also noticeable, which was higher in men than women. In a similar study, pelvic and lower limb fractures were the most common causes of disability up to one year after MVAs^41^, which was the most common type of concurrent injury reported in our injured patients. Head injuries due to PMVAs have been an important cause of the high death rate in recent years while many of these collisions leading to brain damage have been predictable and preventable. Reports have shown that countries that have taken precautionary measures and implemented more safety strategies have been successful in reducing MVAs. Since a lot of costs are imposed on the governments and different levels of society, adopting appropriate preventive measures is of great importance^32^.

## Conclusions

The significant number of PMVAs has shown the dominant pattern of head injuries in male pedestrian residents on the urban streets of Rasht city. In addition, the increasing rate of mortalities and severe disabilities especially in men makes prevention a necessity. A significant number of pedestrians injured in PMVAs had been brought to the hospital by private cars without specialized care. This too highlights the need to reconsider the transfer mode of the injured patients from the scene of the accident to medical centers.

## Abbreviations

MVAs: Motor vehicle accidents
PMVAs: Pedestrian-motor vehicle accidents
EMS: Emergency medical system
ISS: Injury severity score
GOS: Glasgow Outcome Scale
GCS: The Glasgow Coma Scale
LOC: Loss of consciousness
